# Anti-malarial property of steroidal alkaloid conessine isolated from the bark of *Holarrhena antidysenterica*

**DOI:** 10.1186/1475-2875-12-194

**Published:** 2013-06-10

**Authors:** Virendra K Dua, Gaurav Verma, Bikram Singh, Aswathy Rajan, Upma Bagai, Dau Dayal Agarwal, NC Gupta, Sandeep Kumar, Ayushi Rastogi

**Affiliations:** 1 Field Unit, Sector-3, Health Centre, BHEL, National Institute of Malaria Research, Hardwar 249403, India; 2 Natural Plant Products Division, Institute of Himalayan Bioresource Technology, Palampur 176 061, India; 3 Department of Zoology, Punjab University, Chandigarh 160014, India; 4 School of Studies in Chemistry, Jiwaji University, Gwalior 474 011, India

## Abstract

**Background:**

In the face of chronic and emerging resistance of parasites to currently available drugs and constant need for new anti-malarials, natural plant products have been the bastion of anti-malarials for thousands of years. Moreover natural plant products and their derivatives have traditionally been a common source of drugs, and represent more than 30% of the current pharmaceutical market. The present study shows evaluation of anti-malarial effects of compound conessine isolated from plant *Holarrhena antidysenterica* frequently used against malaria in the Garhwal region of north-west Himalaya.

**Methods:**

*In vitro* anti-plasmodial activity of compound was assessed using schizont maturation and parasite lactate dehydrogenase (pLDH) assay. Cytotoxic activities of the examined compound were determined on L-6 cells of rat skeletal muscle myoblast. The four-day test for anti-malarial activity against a chloroquine-sensitive *Plasmodium berghei* NK65 strain in BALB/c mice was used for monitoring *in vivo* activity of compound. In liver and kidney function test, the activity of alkaline phosphatase (ALP) was examined by p-NPP method, bilirubin by Jendrassik and Grof method. The urea percentage was determined by modified Berthelot method and creatinine by alkaline picrate method in serum of mice using ENZOPAK/CHEMPAK reagent kits.

**Results:**

Compound conessine showed *in vitro* anti-plasmodial activity with its IC_50_ value 1.9 μg/ml and 1.3 μg/ml using schizont maturation and pLDH assay respectively. The compound showed cytotoxity IC_50_= 14 μg/ml against L6 cells of rat skeletal muscle myoblast. The isolated compound from plant *H*. *antidysenterica* significantly reduced parasitaemia (at 10 mg/kg exhibited 88.95% parasite inhibition) in *P*. *berghei*-infected mice. Due to slightly toxic nature (cytotoxicity = 14), biochemical analysis (liver and kidney function test) of the serum from mice after administration of conessine were also observed.

**Conclusion:**

The present investigation demonstrates that the compound conessine exhibited substantial anti-malarial property. The isolated compound could be chemically modified to obtain a more potent chemical entity with improved characteristics against malaria.

## Background

Malaria is re-emerging as the world's number one killer infection, causing approximately one million deaths annually and 300–400 million infections per annum [1]. The dreaded disease is difficult to eradicate and its control is possible only with coordinated efforts of the general public, healthcare personnel and government agencies. Dhingra and co-workers [2] challenges the World Health Organization (WHO) observation system; as per WHO more than 10 million malaria cases each year cause 15,000 deaths while based on verbal autopsy investigations between 2001 and 2003, researchers suggest that WHO figures are a huge miscalculate, and the true number is at least 125,000 deaths per year. Moreover exterior Africa, it is the profusely populated Southeast Asia where 30% of the total population is approximated to be at risk of malaria, of which India contributes (80%) most of the cases [3]. The emergence of drug resistance particularly to chloroquine and sulphadoxine-pyrimethamine has led to recommendations that they be replaced with artemisinin-based combination therapy (ACT) for improved efficacy. On the other hand, development of resistance to artemisinins and their associate drugs cruelly limit the utility of ACT in future. Consequently to develop alternative therapy, research on anti-malarials is urgently vital. Plants used in traditional medicines shows potential source of compounds with good anti-malarial activity [4,5]. Natural plant products and their derivatives have traditionally been a common source of drugs, and represent more than 30% of the current pharmaceutical market [6,7].

The continuing research directed towards discovery of anti-malarials from plants [8-10], it was noticed that chloroform extract of *H*. *antidysenterica* showed the anti-malarial properties against *Plasmodium falciparum* isolates and *P. berghei*-infected mice. Therefore, in the present investigation the anti-malarial activity of isolated principle compound conessine from *H*. *antidysenterica* is demonstrated against *P*. *falciparum* isolates and *Plasmodium berghei*-infected mice.

## Methods

### Collection of plants and isolation of compound

**Figure 1:**
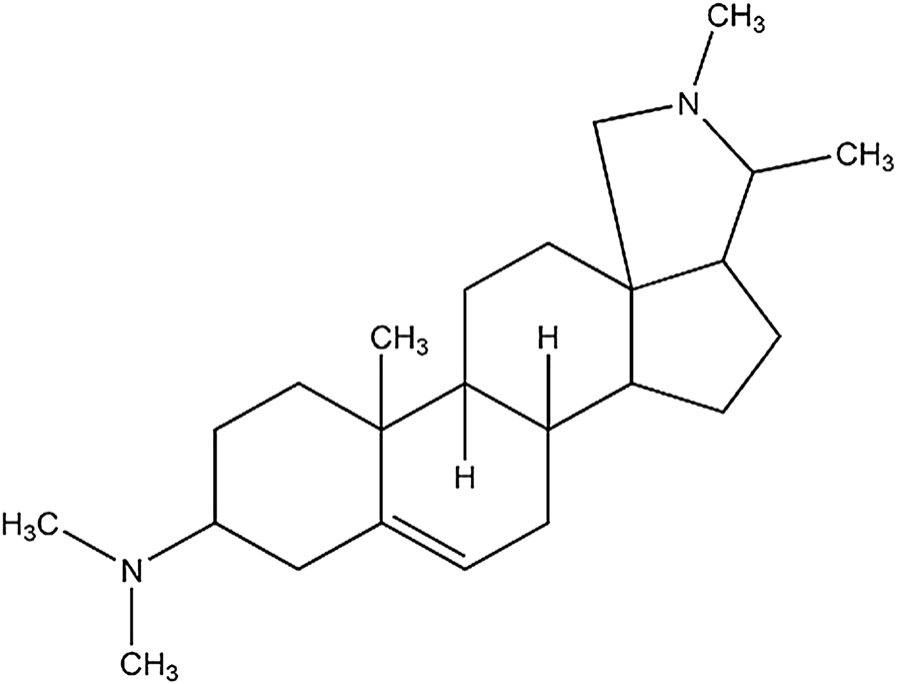
**Structure of steroidal alkaloids conessine isolated from the bark of *Holarrhena antidysenterica.***

Plants and its parts were collected during flowering season of year 2008 from Cheela range of the Garhwal region and identified by Botanical Survey of India, Dehradun, India. Voucher specimens of the plants were stored in the Institute herbarium (voucher specimen number NIMRHAR-101-HA) for future reference. The compound conessine (Figure 1) was isolated from the bark of *H*. *antidysenterica* using the method reported earlier [11].

### *In vitro* anti-plasmodial activity against K1 strain of *plasmodium falciparum* isolates

*In vitro* anti-plasmodial sensitivity of compound was assessed at National Institute of Malaria Research, New Delhi, India using Schizont maturation method [12]. Chloroquine sensitive strain FSG of *P*. *falciparum* derived from an Indian patient of Shahjahanpur (UP) was used for the study. Culture was maintained in A +ve erythrocytes using RPMI 1640 medium supplemented with AB Rh +ve human serum (10%), sodium bicarbonate (0.2%), HEPES buffer (25 mM) and gentamycin (50 μg ml^-1^). The culture was treated with selected concentrations of conessine. The prepared blood smears were stained with Giemsa strain after 72 hrs of incubation % maturation of schizonts against positive control was recorded. The compound was also sent to Swiss Tropical Institute, Switzerland for screening of *in vitro* anti-plasmodial activity using the parasite lactate dehydrogenase (pLDH) assay [13]. In parasite lactate dehydrogenase (pLDH) assay, chloroquine sensitive GHA strain derived from a Ghanaian patient was used and maintained in RPMI 1640 medium supplemented with 25 mM HEPES, 0.37 mM hypoxanthine, 10% A+ve human serum together with 2-4% washed human O +ve erythrocytes and 25 mM NaHCO_3_. All cultures were conducted at 37 ± 1°C and an atmosphere of 3% oxygen, 4% carbon dioxide and 93% nitrogen. The sterile 384- well microtiter plates were used for performing assays, in which each well containing 2 μl of selected concentration of compound solution with 38 μl of the parasite inoculums (1% parasitaemia, 2% haematocrit). Parasite growth was compared to control wells (100% parasite growth). After 72 h of incubation at 37 ± 1°C, plates were deep-frozen at −20°C. After thawing 5 μl from each well was transferred into another plate together with 25 μl of Malstat™ reagent and NBT (Nitro Blue Tetrazolium, 0.1 mg/ml) and 5 μl of a 1/1 mixture of PES (phenazine ethosulfate, 2 mg/ml). The plates were then kept into darkness for 2 h and the change in colour was measured with a spectrophotometer (at 655 nm). In both methods chloroquine was taken as positive control. The inhibitory concentration value, at which 50% of the parasites kill (IC_50_) was considered for anti-plasmodial activity.

### Cytotoxicity on rat skeletal muscle myoblasts (L-6 cells) and selectivity indices (SI)

The cytotoxicity of the compound was determined using reported method [14,15]. The cell line L-6, rat skeletal muscle myoblasts were seeded in 96-well Costar microtiter plates at 2 × 103/cells/100 ml, 50 ml per well in MEM supplemented with 10% heat inactivated FBS. A three-fold serial dilution ranged from 90 to 0.13 mg/ml of compounds in test medium was added. Plates with a final volume of 100 ml per well were incubated at 37±1°C for 72 h in a humidified incubator containing 5% CO_2_ and resazurin was added as viability indicator. After an additional 2 h of incubation, the plates were measured with a fluorescence scanner using an excitation wavelength of 536 nm and an emission wavelength of 588 nm. The IC_50_ values were calculated from the sigmoidal inhibition curves with the SoftmaxPro software.

The selectivity indices (SI) were calculated with the ratio of the IC_50_ for the L-6 cells to the IC_50_ for the *in vitro* anti-plasmodial activity against *P*. *falciparum* isolates.

### *In vivo* anti-malarial activity against *P*. *Berghei*

#### Mouse and parasite strain

BALB/c mice (weighing 22–26 g and 4–6 weeks old) of either sex, obtained from the central animal house, Panjab University, Chandigarh were used as experimental models. They were maintained on a standard pellet diet and water *ad libitum*. *Plasmodium berghei* (NK- 65) was maintained by intraperitoneal inoculation of 1×10^6^ infected red blood cells (RBCs) to native mice [16].

#### Ethical clearance

The treatment of mice was according to the guidelines of committee for the purpose of control and supervision on experiments on animals (Reg No. 45/1999/CPCSEA), Panjab University, Chandigarh, India.

#### Drug used

Compound was dissolved in 70% Tween 80 and 30% ethanol. This solution was further diluted 10-fold with distilled water to result in a stock solution containing 7% Tween and 3% ethanol with which different concentrations of compound, i.e., 10 mg/kg, 20 mg/kg and 50 mg/kg were prepared.

#### Experimental design

Five groups having 10 mice each (same sex and age) were used for the present study. Groups were designated as G-1 to G-5 (Table 1). All groups were injected with 1×10^6^*P*. *berghei* parasitized RBCs on day 0. One to two hours post-infection, the experimental groups were treated with a single dose of test compound. Dose of various compounds/vehicles (0.2 ml/mouse/day) were administered orally to mice of different groups for four days (day 0 to 3) according to Peters four-day test [17] 24 hours after the last treatment (day 4, i.e., day 5), Giemsa-stained blood smears were prepared from tail vein incision and percent reduction of parasite (activity) was calculated in various groups.

$$\text{Activity}\;=\;100-\frac{\text{Mean}\;\text{parasitaemia}\;\mathrm{of}\;\text{treated}}{\text{Mean}\;\text{parasitaemia}\;\mathrm{of}\;\text{control}}\times 100$$

**Table 1:** **Experimental groups for four**-**day suppressive test**

**Group**	**Drug/Vehicle**
n=10	0.2 ml/mouse/day
G1	10 mg/kg
G2	20 mg/kg
G3	50 mg/kg
G4 (Vehicle control)	70% Tween 80 + 30% ethanol
G5 (Infected control)	Distilled water

All the groups was injected with 1×10^6^-infected red blood cells on day 0.

n = number of mice in each group.

The mean survival time (MST) of each group was calculated up to 2 weeks (14 days) post inoculation.

#### Liver and kidney function tests

Adverse effects due to the compound were examined by liver and kidney function tests. The activity of alkaline phosphatase (ALP) was determined by p-NPP method [18], bilirubin by Jendrassik and Grof method [19]. The urea concentration was determined using modified Berthelot method [20] and creatinine by alkaline pictrate method [21] in serum of mice using ENZOPAK/CHEMPAK reagent kits (Reckon Diagnostic Pvt Ltd, Gorwa, Baroda, India). Serum was obtained from four mice of each group by centrifugation of blood at 800 g for 15 min RT. Biochemical assays were performed on day 7 in infected control and on day 10 in treated groups.

## Results and discussion

Natural plant products have remained the eventual foundation for the treatment of various ailments, including malaria forever [8,10,22,23]. The chloroform extract of plant *H*. *antidysenterica* was found to wield considerable *in vitro* anti-malarial activity with an IC_50_ value of 5.5 μg/ml against chloroquine-sensitive *P*. *falciparum* isolates, during parasite lactate dehydrogenase (pLDH) assay [9]. Therefore in the present study, the anti-malarial activity of the principal steroidal alkaloid, conessine from the stem bark of *H*. *antidysenterica*, was evaluated. Conessine was isolated from the stem bark using the methods reported earlier [11]. The anti-plasmodial activity, cytotoxic activity and selectivity indices of conessine are presented in Table 2. The *in vitro* anti-plasmodial activity of conessine assessed by pLDH assay revealed more reproducible value (IC_50_ = 1.3 μg/ml and) as compared to the activity assessed by schizont maturation method (IC_50_ = 1.9 μg/ml). The assessed cytotoxity of conessine against a rat cell line L-6 was found IC_50_=14 μg/ml. While Zirihi and co-workers [24] demonstrated *in vitro* anti-plasmodial activity of conessine isolated from the plant *Funtumia elastica* against chloroquine-resistant strain FcB1 of *P*. *falciparum* with its IC_50_ 1.04 μg/ml and cytotoxity against a rat cell line L-6 with 14 μg/ml respectively. Stephenson [25] showed that conessine had no anti-malarial action in chicks infected with *Plasmodium gallinaceum*, while in the present investigation (Table 3), conessine was significantly reduce parasitaemia (Table 3) compared to the activity of the same plant chloroform extracts (70% at 30 mg/kg dose) [9] and the untreated control. It was observed that compound conessine reduce higher percentage of parasitaemia at low dose range (88.9%, at 10 mg/kg dose on day five) as compared to high dose range (reduce 71.6% of parasitaemia at 50 mg/kg dose on day five).Therefore percentage reduction of parasitaemia was further observed on day seven and was found high at low dose range (88.95%, at 10 mg/kg dose on day seven) as compared to high dose range (reduce 50.99% of parasitaemia at 50 mg/kg dose on day seven). Walker and collaborators [26] elaborated that highly potent drug evokes a larger response at low concentration that normally occur shortly after adequate treatment doses but are not killed by suboptimal doses or by drug concentrations later on in the terminal elimination phase. Due to which the activities in all the groups were observed to decrease during the follow up period. After administration of compound conessine, the observed survival of animals (Table 3) supported for its anti-plasmodial activity compared with other steroidal alkaloids [24,27]. Due to the slightly toxic nature (cytotoxicity = 14 μg/ml), biochemical analysis (liver function tests and kidney function test) of the serum of the mice after administration of conessine was also observed and is summarized in Table 4. In terms of pathogenesis, the host liver is among the organs affected in the early stage of malaria [28] leading to significant alterations in the host hepatocyte physiology and morphology. Elevated levels of ALP and bilirubin are an indication of hepatocyte damage due to malarial infection [29]. Acute renal failure or acute kidney injury and increased levels of creatinine and urea have been also associated with severe falciparum and vivax malaria [30]. Biochemical analysis of uninfected mice administered with the compound showed that conessine affected the function of the liver and kidney significantly as compared to normal mice (Table 4). In earlier studies, chloroquine has also been reported to induce secondary increase in the certain enzymatic activities along with the increase in autophagy of cells exposed to chloroquine. Hence, this could probably justify the observed elevation in enzyme activity of liver and kidney of treated mice [31]. The administration of artemisinin is also reported to cause a significant increase in serum aspartate aminotransferase, ALP and alanine amino transferase activities [32]. The increased enzymatic activities in the study suggest that the extract might have affected the hepatic and renal indices without producing cellular necrosis. An increased level of various biochemical parameters has been reported due to administration of various anti-malarial drugs. Conessine exhibited strong anti-plasmodial activity during the course of infection as compared to the vehicle and infected control mice (Table 3). Results of the suppressive activity exhibited a good clearance rate at low concentration of 10 mg/kg (88.95%) (Table 3).

**Table 2:** ***In vitro *anti**-**plasmodial activity of examined compound**

**Compound**	**Anti**-**plasmodial activity (IC**_**50 **_**μg/ml)**		**Cytotoxity**	**Selectivity indices**	
	**A**	**B**	**IC**_**50 **_**(μg/ml)**	**A**	**B**
Conessine	1.9	1.3	14	7	10
Chloroquine	0.07	0.051	-	-	-

A: schizont maturation Method; B: pLDH method; cytotoxicity IC_50_ (As per WHO criteria) >16 μg/ml- non cytotoxic; selectivity indices = ratio of IC_50_ of cytotoxic activity/IC_50_ of anti-plasmodial activity [8].

**Table 3:** Course of parasitaemia after inoculation of compound in different experimental groups

**Group**	**Day 5**		**Day 7**		**MST (days)**	**% survival of animals on day 14**
	**Parasitaemia (%)**	**Activity (%)**	**Parasitaemia (%)**	**Activity (%)**		
**G1**	0.8 ± 0.02	88.9	3.36 ± 2.1*	88.95	13.3	40
**G2**	2.15 ± 0.4	70.2	13.2 ± 2*	56.6	11.6	20
**G3**	2.05 ± 1	71.6	14.9 ± 3.12*	50.99	11.5	20
**G4**	6.6 ± 2.4	8.4	28.7 ± 4.5^NS^	5.6	7	
**G5**	7.2 ± 1.8		30.4 ± 3.6		7.75	

Data is given as mean ± SD. Significance of values on day 7 were calculated in comparison to infected control and p-value<0.0005 is marked as *=extremely statistically significant, NS=not significant, MST=Mean Survival Time.

**Table 4:** **Alkaline phosphatase** (**ALP**) **activity**, **concentration of bilirubin**, **urea and creatinine in different experimental groups on day 10 post inoculation of compound**

**Group**	**ALP (KA units)**	**Bilirubin (mg/dl)**	**Urea (mg/dl)**	**Creatinine (mg/dl)**
**G1**	11.5±1.3^NS^	1.3 ± 0.5**	160 ± 4.3**	8.55 ± 1.4**
**G2**	15.3 ± 1.4*	2.6 ± 0.84*	176 ± 2.8**	8.5 ± 2.2**
**G3**	19.2 ± 3.3**	1.3 ± 0.02**	112 ± 4.22**	10 ± 2.7**
**G5**	18.33 ± 2.11**	1.31 ± 0.02**	147.5 ± 2.1**	1.8 ± 0.03**
**Normal mice**	11 ± 2.3	1.05 ± 0.1	38.26 ± 2.6	0.83 ± 0.1

Note: Data is given as mean ± SD. Significance of values were calculated in comparison to normal control and p-value<0.005 is marked as *=very statistically significant, p-value<0.0005 **=extremely statistically significant, *NS* not significant.

## Conclusion

The steroidal alkaloid conessine isolated from the bark of *H*. *antidysentrica* exhibited substantial anti-malarial activity with slight cytotoxic nature. The isolated compound could be chemically modified to obtain a more potent chemical entity with improved characteristics or modified compound can become preclinical candidate against malaria.

## Competing interests

The authors declare that they have no competing interests.

## Authors' contributions

GV (PhD student) contributed in the laboratory work, analysis of the data and drafted the paper. VKD, BS and DDA designed the work and supervised the *in vitro* studies, analysed the data and prepared the paper. UB and AR designed and performed the *in vivo* studies as well as liver and kidney function tests. NCG, SK and AR contributed in the laboratory work. All authors contributed to the critical review of the manuscript and agree to submission.
